# Multicenter Study on Integrating Prostate Magnetic Resonance Imaging with Prostate-Specific Antigen Density for Risk-Adapted Biopsy Strategy in a South Korean Cohort

**DOI:** 10.5152/tud.2026.26014

**Published:** 2026-05-13

**Authors:** Seung-Ju Lee, Dong Ho Shin, Hee Youn Kim

**Affiliations:** 1 Department of Urology, St. Vincent’s Hospital, The Catholic University of Korea College of Medicine, Seoul, Republic of Korea; 2 Department of Urology, Seoul St. Mary's Hospital, The Catholic University of Korea College of Medicine, Seoul, Republic of Korea

**Keywords:** Biopsy, magnetic resonance imaging, prostate cancer

## Abstract

**Objective:**

Combining Prostate Imaging-Reporting and Data System (PI-RADS) with prostate-specific antigen density (PSAD) may improve the detection of clinically significant prostate cancer (csPCa) while reducing unnecessary biopsies. This study aimed to evaluate csPCa detection rates using PI-RADS and PSAD, identify optimal PSAD cut-offs, and assess biopsy strategies to optimize csPCa detection and reduce unnecessary procedures within a South Korean cohort.

**Methods:**

This multicenter retrospective study included 3117 biopsy-naïve patients from 2 tertiary hospitals in South Korea (2020-2025) who underwent magnetic resonance imaging–based transperineal prostate biopsy. Patients were stratified into PI-RADS groups (1-2, 3, 4-5) and PSAD categories (<0.10, 0.10-0.15, 0.15-0.20, and ≥0.20). Receiver-operating characteristic (ROC) curve analyses validated PSAD cut-offs, and biopsy strategies were compared for csPCa detection and biopsy avoidance.

**Results:**

The overall csPCa detection rate was 47.1%. PI-RADS 4-5 patients had high detection rates across all PSAD levels (20.1%-76.5%), while PI-RADS 1-2 and 3 patients with PSAD ≥ 0.15 showed elevated rates (15.2%-16.9% and 25.0%-35.7%, respectively). ROC curve analyses identified optimal PSAD cut-offs of 0.155 (area under the ROC curve (AUC), 0.708) for PI-RADS 1-2 and 0.145 (AUC, 0.749) for PI-RADS 3. The proposed strategy (PI-RADS ≥ 4 or PI-RADS 1-2 or 3 with PSAD ≥ 0.15) outperformed other strategies, avoiding 448 (14.4%) biopsies, missing 28 (1.9%) csPCa cases, and achieving a negative predictive value of 93.8%.

**Conclusion:**

Integrating PI-RADS with PSAD enhances risk stratification for csPCa, maintaining high diagnostic accuracy while reducing unnecessary procedures.

**Main Points**

- Enhanced risk stratification: Integrating prostate-specific antigen density (PSAD) with Prostate Imaging-Reporting and Data System (PI-RADS) scores significantly enhances the diagnosis of clinically significant prostate cancer (csPCa), maintaining high diagnostic accuracy while reducing unnecessary procedures in biopsy-naïve patients.
- Specific biopsy criteria: Patients with PI-RADS 4-5 warrant biopsy regardless of PSAD due to high detection rates, whereas a PSAD threshold of ≥ 0.15 ng/mL^2^ was identified as the optimal cut-off for recommending biopsy in PI-RADS 1-3 lesions.
- Comparison to conventional guideline: The proposed risk-adapted strategy (Biopsy for PI-RADS ≥ 4, or PI-RADS 1-3 with PSAD ≥ 0.15) avoided 14.4% of biopsies, missed 1.9% of csPCa cases, with a negative predictive value of 93.8%, outperforming the 2025 European Association of Urology guideline recommendations in this cohort.
- Population-specific validation: Validating these parameters in an East Asian cohort underscores the importance of tailoring biopsy strategies to population-specific cancer prevalence, which was higher (47.1%) in this study compared to typical Western cohorts.

## Introduction

Pre-biopsy multiparametric prostate magnetic resonance imaging (MRI) and use of the Prostate Imaging-Reporting and Data System (PI-RADS) have significantly improved the detection of clinically significant prostate cancer (csPCa) while reducing the diagnoses of insignificant prostate cancer and unnecessary biopsies.^1^^-^^5^ As a result, major clinical guidelines recommend pre-biopsy MRI for prostate cancer diagnosis.^6^^,^^7^

To further refine risk stratification in patients undergoing pre-biopsy MRI, several studies have investigated the combined use of PI-RADS and prostate-specific antigen density (PSAD), both of which are independent predictors of csPCa.^8^^,^^9^ These studies have demonstrated that incorporating PSAD into MRI-based biopsy decision-making could help avoid a substantial proportion of unnecessary biopsies while maintaining an acceptable detection rate of csPCa.^10^^,^^11^ However, given variations in prostate cancer prevalence across different populations, external validation in diverse cohorts is essential.^11^

The objectives of this study are (1) to evaluate the detection rate of csPCa based on PI-RADS and PSAD, (2) to identify optimal PSAD cut-off points, (3) to compare the findings with those of previous studies conducted in different and similar populations, and (4) to compare biopsy strategies applied to the cohort to enhance risk-adapted decision-making for csPCa detection and biopsy avoidance.

## Material and Methods

### Study Population and Design

This multicenter retrospective study was conducted using medical records from 2 high-volume tertiary hospitals in South Korea (Seoul St. Mary’s Hospital and St. Vincent’s Hospital). between November 2020 and February 2025. The study was approved by the Institutional Review Board (IRB) of both participating hospitals (IRB no. VC23OISI0255) on May 2, 2025. A waiver for consent was obtained due to the retrospective nature of the study. Biopsy-naïve patients who underwent MRI-based transperineal prostate biopsy were identified. Patients were included if they had available pre-biopsy multiparametric MRI results. Patients were excluded if they were missing essential data or had a history of focal therapy prior to biopsy. The following data were retrospectively collected: age, PSA level (ng/mL), PI-RADS score (1-5 points), prostate volume (mL), PSAD (ng/mL^2^), number of biopsy cores, and histopathologic findings (Gleason score and International Society of Urological Pathology grade group in case of malignancy). Digital rectal examination findings were excluded from the current analysis due to inconsistent documentation across the participating institutions. Multiparametric MRI was evaluated using PI-RADS, version 2.1^12^ by dedicated genitourinary radiologists, who have >5 years of experience with PI-RADS scoring and have evaluated a high institutional volume of approximately 800 prostate MRIs annually. Prostate volume was determined from MRI, as MRI-based measurements provide greater accuracy than transrectal ultrasound images.^13^ PSAD was calculated as PSA (ng/mL) divided by prostate volume (mL). csPCa was defined as Gleason grade group ≥2. All patients underwent MRI-based transperineal prostate biopsy, either via MRI-ultrasound fusion biopsy or cognitive biopsy, based on clinician discretion. The detailed biopsy procedure is described in previous publications.^14^^,^^15^ The biopsy strategy was as follows: systemic biopsy only in patients with PI-RADS 1-2, targeted biopsy combined with systematic biopsy in patients with PI-RADS 3-5, and targeted-only biopsy in selected cases of locally advanced disease.

This study used Gemini/Google to improve the readability and language during the preparation of the manuscript. However, all content of the study has been reviewed and edited by the author following the use of AI/LLM tools.

### Statistical Analysis

Baseline patient characteristics are summarized using descriptive statistics. Continuous variables are presented as median with Q1 and Q3 values, while categorical variables are reported as percentages (%). Patients were categorized into 3 PI-RADS groups (1-2, 3, and 4-5) and further stratified into 4 PSAD groups (<0.10, 0.10-0.15, 0.15-0.20, and ≥0.20). A cross-tabulation table was constructed to assess the diagnostic rate of csPCa in each PI-RADS and PSAD combination.

To validate the PSAD cut-off value identified in the cross-tabulation analysis, separate receiver-operating characteristic (ROC) curve analyses were performed for PI-RADS subgroups 1-2 and 3. The area under the ROC curve (AUC) was calculated to assess the diagnostic performance. The optimal PSAD cut-off values were explicitly determined using Youden’s Index (sensitivity + specificity − 1). Youden’s Index identifies the threshold that mathematically maximizes the overall diagnostic accuracy, providing an objective balance between maximizing the detection of csPCa (sensitivity) and minimizing unnecessary biopsies (specificity). Additionally, sensitivity, specificity, positive predictive value (PPV), and negative predictive value (NPV) at these optimal cut-offs were determined. All statistical analyses were conducted using SPSS, version 30.0 (IBM Corporation, Armonk, NY, USA). *P*-value <.05 was considered statistically significant.

## Results

Table 1 describes the baseline characteristics of the study population. A total of 3117 biopsy-naïve patients were included in the study. The median age was 68 years, and the median PSA level was 7.0 ng/mL. The majority of patients (69.5%) had a PSA level of <10. The median prostate volume was 39.3 mL, and the median PSAD was 0.18. The overall detection rate of csPCa was 47.1%. Comparisons between csPCa and non-csPCa patients are presented, with corresponding statistical tests reported. A subgroup analysis to clarify the effect of age and PSA on detection rate of csPCa was performed (Supplementary Table 1). The results showed that the cohort’s overall detection rate was driven by older age (65.3% for men >75 years) and higher PSA (85.4% for PSA >20 ng/mL).

Table 2 presents a cross-tabulation analysis of the diagnosis rate of csPCa based on PI-RADS score and PSAD. PI-RADS 4-5 patients had a high csPCa detection rate across all PSAD groups, ranging from 20.1% in PSAD <0.10 to 76.5% in PSAD ≥0.20, indicating that biopsy should be performed regardless of PSAD. PI-RADS 3 patients showed a marked increase in csPCa detection rate above PSAD 0.15; csPCa detection rates were 25.0% and 35.7% in PSAD 0.15-0.20 and ≥0.20, respectively, but only 4.1% and 9.9% in PSAD <0.10 and 0.10-0.15, respectively. PI-RADS 1-2 patients demonstrated a similar trend; csPCa detection rates were 15.2% and 16.9% in PSAD 0.15-0.20 and ≥0.20, respectively, whereas they were 2.6% and 7.5% in PSAD <0.10 and 0.10-0.15, respectively. These results suggest a PSAD threshold of 0.15 to be an appropriate cutoff value for biopsy decision in PI-RADS 1-2 and 3 patients.

**Figure 1:**
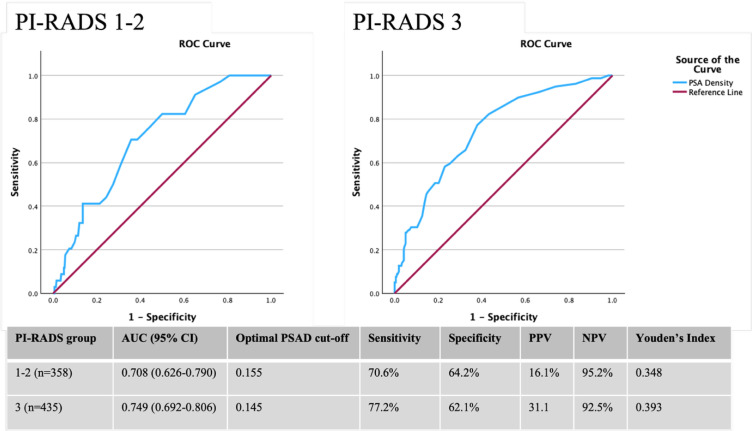
Receiver-operating characteristic (ROC) curve analysis of prostate-specific antigen (PSA) density for clinically significant prostate cancer in Prostate Imaging-Reporting and Data System (PI-RADS) 1-2 and 3 groups. The graphs illustrate the diagnostic performance of prostate-specific antigen density (PSAD) in patients classified as PI-RADS 1-2 (left) and PI-RADS 3 (right). The blue line represents the PSAD performance, while the red line represents the reference. The accompanying table summarizes the area under the curve (AUC) with 95% CI, optimal PSAD cut-off values determined by Youden’s Index, and the corresponding sensitivity, specificity, positive predictive value (PPV), and negative predictive value (NPV) for each subgroup.

To validate the PSAD cut-off value identified in the cross-tabulation analysis, separate ROC curve analyses were performed for PI-RADS 1-2 and 3 patients (Figure 1). In PI-RADS 1-2 patients (n = 358), ROC analysis of PSAD for predicting csPCa yielded an AUC of 0.708 (95% CI), 0.626-0.790, *P* < .001). The optimal PSAD cut-off was 0.155 (Youden’s Index, 0.348), with a sensitivity of 70.6%, specificity of 64.2%, PPV of 16.1%, and NPV of 95.2%. For PI-RADS 3 patients (n = 435), the AUC was 0.749 (95% CI, 0.692-0.806, *P* < 0.001), with an optimal PSAD cutoff of 0.145 (Youden’s Index, 0.393), corresponding to a sensitivity of 77.2%, specificity of 62.1%, PPV of 31.1%, and NPV of 92.5%. These findings support a PSAD threshold of ≥0.15 for identifying csPCa across both PI-RADS 1-2 and 3 categories. The full ROC curve coordinates for PI-RADS 1-2 and PI-RADS 3 groups are provided in Supplementary Table 2.

Based on the study’s findings, a risk-adapted prostate biopsy strategy for csPCa detection is proposed, as outlined in Table 3. To categorize csPCa detection rates and guide biopsy recommendations, the risk stratification system proposed by Schoots and Padhani was adopted, which defines risk levels based on detection rates and corresponding biopsy actions (Supplementary Table 3).^11^ For PI-RADS 1-2 patients, no biopsy is recommended for PSAD <0.15 (very low to low risk), whereas biopsy should be considered for PSAD ≥0.15 (intermediate-low risk). For PI-RADS 3 patients, no biopsy is recommended for PSAD <0.15 (very low to low risk), with a high consideration for biopsy at PSAD 0.15-0.20 (intermediate-high risk), and biopsy is recommended for PSAD ≥0.20 (high risk). For PI-RADS 4-5 patients, biopsy is recommended across all PSAD levels due to consistently high detection rates (intermediate-high to very high risk).

Table 4 evaluates the performance of different biopsy strategies applied to the cohort of 3117 patients, assessing biopsy avoidance, missed csPCa, sensitivity, specificity, PPV, and NPV, with transperineal prostate biopsy results confirming csPCa presence. Compared to using PI-RADS scores alone, adding PSAD significantly improves patient stratification: biopsying all PI-RADS ≥4 patients avoided 793/3117 biopsies (25.4%) but missed 113/1467 csPCa cases (7.7%), while biopsying all PI-RADS ≥3 patients reduced missed csPCa to 34/1467 (2.3%) but avoided fewer biopsies (358/3117, 11.5%). Incorporating PSAD allows for more nuanced risk stratification, particularly in PI-RADS 1-2 and 3 patients, where a PSAD ≥0.15 threshold identifies increased-risk individuals while avoiding unnecessary procedures in low-risk groups. The proposed strategy (PI-RADS ≥ 4, PI-RADS 3 with PSAD ≥ 0.15, PI-RADS 1-2 with PSAD ≥0.15) outperformed the recommendations from the 2025 EAU guideline^6^ (PI-RADS ≥ 4, PI-RADS 3 with PSAD ≥ 0.10, PI-RADS 1-2 with PSAD ≥ 0.20), avoiding 448/3117 biopsies (14.4%) compared to 372/3117 (11.9%)–a 20% relative improvement–while missing only 28/1467 csPCa cases (1.9%) vs. 24/1467 (1.6%). Additionally, this strategy achieved a greater sensitivity (98.1% vs. 97.4%), specificity (25.5% vs. 21.1%), PPV (53.9% vs. 52.3%), and NPV (93.8% vs. 90.1%), demonstrating its potential to optimize biopsy decisions while maintaining high csPCa detection rates. 

To further evaluate the clinical safety of this proposed strategy, the characteristics of the 28 missed csPCa cases out of the 448 avoided biopsies were analyzed. The majority of the missed csPCa cases were Gleason grade group 2, with 19 cases (67.9%). Of the remaining missed cases, 5 (17.9%) were Gleason grade group 3, 3 (10.7%) were Gleason grade group 4, and 1 (3.6%) was Gleason grade group 5. To contextualize the clinical trade-off across different biopsy strategies, the number needed to biopsy (NNB) to detect 1 missed csPCa case within the respective biopsy strategies was calculated. The NNB was 7 when biopsying PI-RADS ≥4 patients, 11 for PI-RADS ≥3, 16 for the 2025 EAU Guideline recommendations, and 16 for the proposed strategy. A higher NNB indicates a lower concentration of undetected cancer.

## Discussion

The current multicenter study of 3117 biopsy-naïve patients demonstrates that combining PI-RADS scores with PSAD enhances the diagnosis of csPCa, with an overall detection rate of 47.1%. The results confirm that PI-RADS 4-5 patients warrant biopsy regardless of PSAD due to high detection rates. For PI-RADS 1-2 and 3 patients with PSAD ≥ 0.15, detection rates were elevated at 15.2%-16.9% and 25.0%-35.7%, respectively, and validated by ROC analyses with optimal cut-offs of 0.155 for PI-RADS 1-2 and 0.145 for PI-RADS 3. The proposed risk-adapted biopsy strategy (PI-RADS ≥4, PI-RADS 3 with PSAD ≥0.15, PI-RADS 1-2 with PSAD ≥0.15) avoided 14.4% of biopsies while missing only 1.9% of csPCa cases, the majority of which were Gleason grade group 2 cancers. These findings underscore the utility of a PSAD ≥0.15 threshold in optimizing csPCa detection while minimizing unnecessary procedures in our population.

The proposed biopsy strategy outperformed the recommendations from the 2025 EAU guideline, which avoided 11.9% of biopsies and missed 1.6% of csPCa cases, by a relative improvement of 20% in biopsy avoidance (absolute difference of 2.5%) and achieved a higher NPV (93.8% vs. 90.1%).^6^ Furthermore calculating the NNB to detect 1 missed csPCa demonstrated that this proposed biopsy strategy achieved the same safety threshold as the EAU guideline of requiring 16 biopsies to find 1 csPCa, while successfully avoiding unnecessary biopsies in a significantly larger number of patients (448 vs. 372). Although a 2.5% absolute improvement in biopsy avoidance may seem modest statistically, this difference becomes highly significant at a population level. In a real-world clinical context, avoiding these procedures translates directly to substantial cost savings for the healthcare system. More importantly, it spares patients from biopsy-related morbidities, including pain, hematuria, urinary retention, and the risk of potentially severe infectious complications such as sepsis. Therefore, maximizing biopsy avoidance while maintaining diagnostic safety carries profound clinical and economic value.

The findings are consistent with those of prior studies demonstrating the value of combining PSAD with MRI-based assessments to optimize the detection of csPCa in biopsy-naïve patients while reducing unnecessary biopsies. Boesen et al found that restricting biopsies to biopsy-naïve men with highly suspicious biparametric MRIs (score ≥4) or PSAD ≥0.15 reduced biopsies by 41% while missing only 5% of csPCa cases.^16^ Stevens et al further corroborated the utility of PSAD with PI-RADS, showing improved predictive performance in men without known prostate cancer, reducing csPCa rates in those with PI-RADS ≤3 from 9.8% to 5.6% at a PSAD threshold of 0.15.^17^ These results are further supported by a systematic review and meta-analysis by Pagniez et al, which reported that a PSAD threshold of <0.15, when paired with negative prebiopsy MRI, increased the NPV from 84.4% to 90.4% in cancer-naïve patients.^18^ Collectively, these studies reinforce the effectiveness of integrating PSAD with MRI-based risk stratification, as demonstrated in the multicenter cohort, to enhance diagnostic accuracy and minimize procedural burden in prostate cancer diagnosis.

Different results were reported from other studies. While this study and that of Schoots and Padhani both employ a risk-adapted approach combining PI-RADS scores with PSAD stratified into risk groups to guide biopsy decisions in biopsy-naïve men, differences in PSAD thresholds for initiating biopsy and detection rates highlight the impact of population variability. The study applies a PSAD threshold of ≥0.15 to start recommending biopsy for both PI-RADS 1-2 and PI-RADS 3 patients, with detection rates of 9.5% for PI-RADS 1-2 (ranging from 2.6% to 16.9%) and 18.2% for PI-RADS 3 (ranging from 4.1% to 35.7%), reflecting an overall csPCa detection rate of 47.1%. In contrast, results from Schoots and Padhani, which are reflected in the 2025 EAU guideline, recommend a PSAD threshold of ≥0.20 to initiate biopsy for PI-RADS 1-2 and a lower threshold of ≥0.10 for PI-RADS 3, with detection rates of 6% for PI-RADS 1-2 (3% to 29%) and 11% for PI-RADS 3 (3% to 77%), and a mean csPCa prevalence of 35%, indicating a population with a lower disease burden.^11^ These discrepancies underscore that there can be no universal PSAD cut-off points for biopsy decisions. Recommendations must be tailored to specific populations with varying race, cancer prevalence, and clinical profiles, a point also emphasized by Schoots and Padhani in their study limitations, which call for validation in diverse populations.^11^ This highlights the need for localized risk stratification strategies to optimize diagnostic accuracy and procedural efficiency in prostate cancer evaluation.

The study’s findings in a South Korean cohort are further supported by studies in similar East Asian populations, where the prevalence of prostate cancer and its diagnostic characteristics may differ from those in Caucasian populations. Washino et al and Konishi et al, analyzing the same cohort of 411 biopsy-naïve Japanese patients with different focuses, reported csPCa detection rates of 49% and 55%, respectively, closely aligning with the detection rate of 47.1%, suggesting a comparable disease burden in East Asian men.^9,19^ Similar to this approach, their study combined biparametric MRI with PSAD, using a threshold of ≥0.15 to recommend biopsy for patients with PI-RADS scores 1-3, and achieved a high NPV of 97% for csPCa when PSAD was <0.15, consistent with the NPV of 93.8%. Additionally, their follow-up data showed that patients with PI-RADS 1-3 and PSAD <0.15 had a low incidence of csPCa detection (3.2% at 36 months), aligning with ther low miss rate of 1.9% and supporting the strategy’s effectiveness in safely avoiding biopsies in low-risk East Asian patients, as evidenced by the 14.4% biopsy-avoidance rate. These similarities underscore the applicability of the risk stratification strategy combining PI-RADS and PSAD in East Asian populations, where tailored diagnostic approaches are crucial due to the relative scarcity of region-specific data compared to Caucasian cohorts.

It is important to clarify that the study does not propose PSAD ≥0.15 as a universal criterion for biopsy decisions but rather demonstrates that incorporating PSAD with PI-RADS enhances risk stratification for csPCa compared to using PI-RADS alone. In addition, rather than adopting a single reference point for biopsy decisions, a shared decision-making approach with patients is advocated, where individual risk profiles, preferences, and clinical contexts are considered to balance the benefits of detecting significant disease against the risks of unnecessary procedures. This patient-centered strategy ensures that diagnostic decisions are tailored to each individual, optimizing both accuracy and patient satisfaction in prostate cancer evaluation.

Several limitations warrant consideration. First, the retrospective design of the analysis may introduce selection bias and limit the ability to control for confounding variables, potentially affecting the generalizability of the findings. Second, the decision to perform a biopsy was left to the discretion of the treating physician, which may have led to variability in biopsy criteria across patients and centers, potentially influencing the observed detection rates and outcomes. Third, although PI-RADS v2.1 was universally applied in the study, MRI reader variability was not accounted for, which is considered moderate at best.^19^ However, all MRIs were interpreted by dedicated genitourinary radiologists with >5 years of PI-RADS experience and a high institutional volume of roughly 800 cases annually, which likely mitigated some of this variability. Future prospective studies with standardized biopsy criteria and assessments of inter-reader variability are needed to validate the findings and further refine the application of PSAD and PI-RADS in risk stratification for prostate cancer.

In conclusion, this multicenter study shows that integrating PI-RADS scores with PSAD enhances risk stratification for csPCa in biopsy-naïve patients, maintaining high diagnostic accuracy while reducing unnecessary procedures. Population variability underscores the need for tailored strategies rather than a universal PSAD threshold. Future prospective studies with standardized biopsy criteria are needed to validate and refine this approach for broader applicability across diverse populations.

## Supplementary Materials

Supplementary Material

## Figures and Tables

**Table 1.** Baseline Characteristics of the Study Cohort and Comparisons Between Non-csPCa and csPCa Patients

	**Whole cohort**	**Non-csPCa**	**csPCa**	*P*
n	3117	1650	1467	
Age (years)	68 (63-74)	66 (62-72)	71 (65-76)	<.001^a^
PSA (ng/mL)	7.0 (5.0-11.4)	6.1 (4.5-8.7)	8.9 (5.8-17.5)	<.001^a^
PSA group <10 10-20 >20	2167 (69.5) 580 (18.6) 370 (11.9)	1347 (81.6) 249 (15.1) 54 (3.3)	820 (55.9) 331 (22.6) 316 (21.5)	<.001^b^
Prostate size (mL)	39.3 (29.5-53.0)	44.2 (34.0-60.2)	34.9 (26.5-44.5)	<.001^a^
PSAD (ng/mL^2^)	0.18 (0.12-0.31)	0.14 (0.09-0.20)	0.27 (0.17-0.52)	<.001^a^
PI-RADS 1-2 3 4-5	358 (11.5) 435 (14.0) 2324 (74.6)	324 (19.6) 356 (21.6) 970 (58.8)	34 (2.3) 79 (5.4) 1354 (92.3)	<.001^b^
Biopsy cores	15 (15-20)	15 (15-20)	15 (13-16)	<.001^a^
csCDR (%)	1467 (47.1)			

csCDR, clinically significant cancer detection rate; PI-RADS, Prostate Imaging-Reporting and Data System; PSA, prostate specific antigen; PSAD, prostate specific antigen density.

Continuous variables: median (Q1-Q3); Categorical variables: n (%)

^a^Mann–Whitney *U*-test.

^b^Chi-square test.

**Table 2.** Diagnosis Rate of Clinically Significant Prostate Cancer Based on PI-RADS Score and PSA Density in Biopsy-Naïve Patients

	**PSA Density**				
PI-RADS	<0.10 (n = 487)	0.10-0.15 (n = 670)	0.15-0.20 (n = 552)	0.20 (n = 1408)	All (n = 3117)
1-2 (n = 358) (%)	3/116 (2.6)	7/93 (7.5)	10/66 (15.2)	14/83 (16.9)	34/358 (9.5)
3 (n = 435) (%)	4/97 (4.1)	14/142 (9.9)	21/84 (25.0)	40/112 (35.7)	79/435 (18.2)
4-5 (n = 2324) (%)	55/274 (20.1)	168/435 (38.6)	203/402 (50.5)	928/1213 (76.5)	1354/2324 (58.3)
All (n = 3117) (%)	62/487 (12.7)	189/670 (28.2)	234/552 (42.4)	982/1408 (69.7)	1467/3117 (47.1)

PI-RADS, Prostate Imaging-Reporting and Data System; PSA, prostate specific antigen.

**Table 3.** Recommended Biopsy Strategy Based on Prostate Cancer Risk

	**PSAD <0.10**	**PSAD 0.10-0.15**	**PSAD 0.15-0.20**	**PSAD ≥0.20**
PI-RADS 1-2	No biopsy	No biopsy	Consider biopsy	Consider biopsy
PI-RADS 3	No biopsy	No biopsy	Highly consider biopsy	Perform biopsy
PI-RADS 4-5	Highly consider biopsy	Perform biopsy	Perform biopsy	Perform biopsy

PI-RADS, Prostate Imaging-Reporting and Data System; PSAD, prostate-specific antigen density.

**Table 4.** Performance of Different Biopsy Strategies Applied to the Population of Current Study

**Biopsy strategy**	**Biopsy avoided (%)**	**Missed csPCa (%)**	**Sensitivity (%)**	**Specificity (%)**	**PPV **(%)****	**NPV **(%)****
PI-RADS ≥4	793/3117 (25.4)	113/1467 (7.7)	92.3	41.2	58.3	85.8
PI-RADS ≥3	358/3117 (11.5)	34/1467 (2.3)	97.7	19.6	51.9	90.5
2025 EAU guideline	372/3117 (11.9)	24/1467 (1.6)	97.4	21.1	52.3	90.1
Current study	448/3117 (14.4)	28/1467 (1.9)	98.1	25.5	53.9	93.8

csPCa, clinically significant prostate cancer; EAU, European Association of Urology; NPV, negative predictive value; PI-RADS, Prostate Imaging-Reporting and Data System; PPV, positive predictive value.

## Data Availability

The data that support the findings of this study are available on request from the corresponding author.

## References

[1] Drost FH Osses DF Nieboer D Prostate MRI, with or without MRI-targeted biopsy, and systematic biopsy for detecting prostate cancer. Cochrane Database Syst Rev. 2019;4(4):CD012663. (doi: 10.1002/14651858.CD012663.pub2) 31022301 PMC6483565

[2] Kasivisvanathan V Rannikko AS Borghi M MRI-targeted or standard biopsy for prostate-cancer diagnosis. N Engl J Med. 2018;378(19):1767 1777. (doi: 10.1056/NEJMoa1801993) 29552975 PMC9084630

[3] Klotz L Chin J Black PC Comparison of multiparametric magnetic resonance imaging-targeted biopsy with systematic transrectal ultrasonography biopsy for biopsy-naive men at risk for prostate cancer: A Phase 3 randomized clinical trial. JAMA Oncol. 2021;7(4):534 542. (doi: 10.1001/jamaoncol.2020.7589) 33538782 PMC7863017

[4] Rouvière O Puech P Renard-Penna R Use of prostate systematic and targeted biopsy on the basis of multiparametric MRI in biopsy-naive patients (MRI-FIRST): a prospective, multicentre, paired diagnostic study. Lancet Oncol. 2019;20(1):100 109. (doi: 10.1016/S1470-2045(18)30569-2) 30470502

[5] van der Leest M Cornel E Israël B Head-to-head comparison of transrectal ultrasound-guided prostate biopsy versus multiparametric prostate resonance imaging with subsequent magnetic resonance-guided biopsy in biopsy-naive men with elevated prostate-specific antigen: a large prospective multicenter clinical study. Eur Urol. 2019;75(4):570 578. (doi: 10.1016/j.eururo.2018.11.023) 30477981

[6] European Association of Urology. EAU guidelines on prostate cancer. Uroweb. Accessed April 9, 2025. https://uroweb.org/guidelines/prostate-cancer.

[7] Wei JT Barocas D Carlsson S Early detection of prostate cancer: AUA/SUO guideline Part II: Considerations for a prostate biopsy. J Urol. 2023;210(1):54 63. (doi: 10.1097/JU.0000000000003492) 37096575 PMC11321723

[8] Distler FA Radtke JP Bonekamp D The value of PSA density in combination with PI-RADS™ for the accuracy of prostate cancer prediction. J Urol. 2017;198(3):575 582. (doi: 10.1016/j.juro.2017.03.130) 28373135

[9] Washino S Okochi T Saito K Combination of prostate imaging reporting and data system (PI-RADS) score and prostate-specific antigen (PSA) density predicts biopsy outcome in prostate biopsy naive patients. BJU Int. 2017;119(2):225 233. (doi: 10.1111/bju.13465) 26935594

[10] Hansen NL Kesch C Barrett T Multicentre evaluation of targeted and systematic biopsies using magnetic resonance and ultrasound image-fusion guided transperineal prostate biopsy in patients with a previous negative biopsy. BJU Int. 2017;120(5):631 638. (doi: 10.1111/bju.13711) 27862869

[11] Schoots IG Padhani AR. Risk-adapted biopsy decision based on prostate magnetic resonance imaging and prostate-specific antigen density for enhanced biopsy avoidance in first prostate cancer diagnostic evaluation. BJU Int. 2021;127(2):175 178. (doi: 10.1111/bju.15277) 33089586 PMC7894174

[12] Turkbey B Rosenkrantz AB Haider MA Prostate imaging reporting and data system. version 2.1; 2019 Update of Prostate imaging reporting and data system version 2.1: 2019 update of prostate imaging reporting and data system version 2. Eur Urol. 2019;76(3):340 351. (doi: 10.1016/j.eururo.2019.02.033) 30898406

[13] Choe S Patel HD Lanzotti N MRI vs transrectal ultrasound to estimate prostate volume and PSAD: impact on prostate cancer detection. Urology. 2023;171:172 178. (doi: 10.1016/j.urology.2022.09.007) 36152871

[14] Kwon HJ Rhew SA Yoon CE Comparing 12-core and 20-core biopsy for prostate cancer diagnosis with transperineal MR/US fusion biopsy: assessing the effective number of systemic cores using propensity score matching. Int Urol Nephrol. 2023;55(10):2465 2471. (doi: 10.1007/s11255-023-03674-2) 37340208 PMC10499967

[15] Lee DS Lee S-J Kim SJ Use versus nonuse of antimicrobial prophylaxis prior to transperineal prostate biopsy: a propensity score-matched analysis. Prostate Int. 2024 [Epub ahead of print];13(2):107 111. (doi: 10.1016/j.prnil.2024.12.002) 40620868 PMC12223518

[16] Boesen L Nørgaard N Løgager V Prebiopsy biparametric magnetic resonance imaging combined with prostate-specific antigen density in detecting and ruling out Gleason 7-10 prostate cancer in biopsy-naive men. Eur Urol Oncol. 2019;2(3):311 319. (doi: 10.1016/j.euo.2018.09.001) 31200846

[17] Stevens E Truong M Bullen JA Clinical utility of PSAD combined with PI-RADS category for the detection of clinically significant prostate cancer. Urol Oncol. 2020;38(11):846.e9 846.e16. (doi: 10.1016/j.urolonc.2020.05.024) 32576527

[18] Pagniez MA Kasivisvanathan V Puech P Predictive factors of missed clinically significant prostate cancers in men with negative magnetic resonance imaging: A systematic review and meta-analysis. J Urol. 2020;204(1):24 32. (doi: 10.1097/JU.0000000000000757) 31967522

[19] Di Franco F Souchon R Crouzet S Characterization of high-grade prostate cancer at multiparametric MRI: assessment of PI-RADS version 2.1 and version 2 descriptors across 21 readers with varying experience (MULTI study). Insights Imaging. 2023;14(1):49. (doi: 10.1186/s13244-023-01391-z) PMC10027981 36939970

