# Supplementary material for: Multicenter Study on Integrating Prostate Magnetic Resonance Imaging with Prostate-Specific Antigen Density for Risk-Adapted Biopsy Strategy in a South Korean Cohort
Source: Urol Res Pract. 2026 May 13;52:e26014. doi: 10.5152/tud.2026.26014 (PMC13242083; doi:10.5152/tud.2026.26014)
Supplement: Supplementary Material [file supplementary_material.pdf]

Supplementary Table 1. Subgroup Analysis of Clinically Significant Prostate Cancer (csPCa) Detection Rates Stratified by Baseline PSA and Age

Variable	Number of patients (total n = 3117)	Number csPCa Diagnosis (total n = 1467)	CDR (overall 47.1%)
Age Group			
<65	984	326	33.1%
65-75	1383	651	47.1%
>75	750	490	65.3%
PSA Group			
<10	2167	820	37.8%
10-20	580	331	57.1%
>20	370	316	85.4%

CDR, cancer detection rate.

Supplementary Table 2. Youden's Index and Corresponding Sensitivity and Specificity Across PSA Density Cut-off Points for PI-RADS 1-2 and 3 Groups

Coordinates of the ROC Curve							
**PI-RADS 1-2 Group**				**PI-RADS 3 Group**			
**Positive if Greater Than or Equal To[a]**	**Sensitivity**	**1 - Specificity**	**Youden's Index**	**Positive if Greater Than or Equal To[a]**	**Sensitivity**	**1 - Specificity**	**Youden's Index**
-1.0000	1.000	1.000	.000	-.9900	1.000	1.000	.000
.0100	1.000	.997	.003	.0250	1.000	.997	.003
.0250	1.000	.991	.009	.0450	1.000	.989	.011
.0350	1.000	.985	.015	.0550	1.000	.980	.020
.0450	1.000	.978	.022	.0650	.987	.947	.041
.0550	1.000	.932	.068	.0750	.987	.904	.083
.0650	1.000	.883	.117	.0850	.962	.831	.131
.0750	1.000	.809	.191	.0950	.949	.739	.211
.0850	.971	.765	.205	.1050	.924	.663	.261
.0950	.912	.651	.261	.1150	.899	.567	.331
.1050	.824	.605	.219	.1250	.861	.500	.361
.1150	.824	.556	.268	.1350	.823	.433	.390
.1250	.824	.500	.324	.1450	.772	.379	.393
.1350	.765	.444	.320	.1550	.658	.326	.332
.1450	.706	.386	.320	.1650	.633	.292	.341
.1550	.706	.358	.348	.1750	.595	.253	.342
.1650	.588	.309	.280	.1850	.582	.230	.352
.1750	.500	.275	.225	.1950	.506	.202	.304
.1850	.441	.244	.197	.2050	.506	.185	.321
.1950	.412	.213	.199	.2150	.468	.154	.314
.2050	.412	.194	.217	.2250	.456	.146	.310
.2150	.412	.170	.242	.2350	.418	.138	.280
.2250	.412	.148	.264	.2450	.354	.126	.228
.2350	.412	.136	.276	.2550	.304	.104	.200
.2450	.324	.136	.188	.2650	.304	.087	.217
.2550	.324	.120	.203	.2750	.304	.076	.228
.2650	.265	.114	.151	.2850	.291	.067	.224
.2750	.265	.105	.160	.2950	.291	.065	.227
.2850	.235	.099	.137	.3050	.278	.053	.225
.2950	.206	.083	.123	.3150	.278	.051	.228
.3050	.206	.074	.132	.3250	.228	.051	.177
.3200	.176	.056	.121	.3350	.215	.045	.170
.3350	.118	.052	.065	.3450	.203	.042	.160
.3450	.118	.049	.068	.3550	.190	.042	.148
.3550	.088	.049	.039	.3650	.165	.042	.122
.3650	.088	.040	.048	.3850	.152	.042	.110
.3750	.088	.037	.051	.4100	.139	.034	.106
.3900	.059	.034	.025	.4250	.127	.031	.096
.4050	.059	.031	.028	.4450	.127	.028	.098
.4150	.059	.028	.031	.4700	.127	.022	.104
.4300	.059	.025	.034	.4850	.127	.020	.107
.4700	.059	.022	.037	.5100	.101	.020	.082
.5050	.059	.019	.040	.5500	.089	.014	.075
.5500	.059	.015	.043	.5800	.089	.011	.077
.6000	.029	.012	.017	.6100	.076	.008	.068
.7600	.029	.009	.020	.6450	.076	.006	.070
.9350	.029	.006	.023	.6800	.063	.006	.058
1.4450	.000	.006	-.006	.7300	.051	.006	.045
2.1150	.000	.003	-.003	.8350	.051	.003	.048

3.3000	.000	.000	.000	.9300	.051	.000	.051
				1.4450	.038	.000	.038
				3.3100	.025	.000	.025
				50.8250	.013	.000	.013
				97.9700	.000	.000	.000

The test result variable(s): PSA Density has at least one tie between the positive actual state group and the negative actual state group.
a. The smallest cutoff value is the minimum observed test value minus 1, and the largest cutoff value is the maximum observed test value plus 1. All the other cutoff values are the averages of two consecutive ordered observed test values.
These tables display the sensitivity, specificity, and Youden's index for different PSA density cut-off points in PI-RADS 1-2 (left) and PI-RADS 3 (right) groups. The optimal cut-off point was determined based on the highest Youden's index, balancing sensitivity and specificity for clinically significant prostate cancer detection.

Supplementary Table 3. Risk Table

Risk[#]	Detection rate (%)	Biopsy recommendation
Very low	0-5 csPCa	No biopsy
Low	5-10 csPCa	No biopsy
Intermediate-low	10-20 csPCa	Consider biopsy
Intermediate-high	20-30 csPCa	Highly consider biopsy
High	30-40 csPCa	Perform biopsy
Very high	>40 csPCa	Perform biopsy

#Risk table based on Shoots and Padhani, BJU Int 2021; 127:175-8.
